# Sensitivity and specificity of the remote evaluation of therapeutic response in cutaneous leishmaniasis using photographs from a mobile application

**DOI:** 10.4269/ajtmh.22-0164

**Published:** 2022-07-25

**Authors:** Alejandra Maria Del Castillo, Maria del Mar Castro, Alexandra Cossio, Jonny Alejandro García Luna, Domiciano Rincón, Ruth Mabel Castillo, Miguel Darío Prieto, David Esteban Rebellón-Sánchez, Andrés Navarro, Neal Alexander

**Affiliations:** 1 Centro Internacional de Entrenamiento e Investigaciones Médicas (CIDEIM), Cali, Colombia; 2 Universidad Icesi, Cali, Colombia; 3 Grupo i2t, Universidad Icesi, Cali, Colombia

**Keywords:** mHealth, cutaneous leishmaniasis, treatment outcome

## Abstract

Cutaneous leishmaniasis (CL) primarily affects people in remote settings with limited access to health services. mHealth tools offer an opportunity to overcome knowledge gaps about clinical response to treatment. We evaluated the validity of the Guaral+ST mobile application for the remote assessment of therapeutic response in patients with CL, through photographs of lesions captured with the app by community health volunteers (CHV). Patients with confirmed CL were followed at weeks 13 and 26 after completion of treatment to assess therapeutic response in two clinical settings in southwest Colombia. Direct evaluation of lesions performed by an experienced physician was considered the reference standard. Photographs of lesions taken by CHV or nurse assistants with the mobile app, were independently evaluated by three physicians to define clinical response. A summary measure of clinical outcome defined by the three physicians was considered the index test. Sensitivity, specificity, positive and negative predictive values were estimated. Inter-rater reliability (kappa) was calculated. Among 53 participants with CL who had at least one follow-up visit, the sensitivity of therapeutic response evaluation through photographs taken with the Guaral+ST app, compared to direct evaluation by an expert physician, had high validity with sensitivity 100% (95% CI 80.5-100%) and specificity (97.2%, 95% CI 85.5-99.9%). The chance-adjusted agreement (κ) was more than 0.8, which is conventionally characterized as almost perfect. The high accuracy of the remote evaluation of photographs for the assessment of therapeutic response supports the use of mHealth tools for improving access to treatment follow-up for CL.

## Introduction

Cutaneous leishmaniasis (CL) remains a public health concern in Colombia with the second highest incidence in the Americas: 94.23 cases per 100,000 inhabitants in 2019^1^. CL disproportionately affects resource-limited and remote communities. In Colombia 80.8% of cases occur in rural areas^2^ where diagnosis, treatment and follow-up are challenging. Currently, data on therapeutic response to antileishmanial treatments in the Americas is mostly limited to randomized clinical trials^3, 4, 5^. Despite the international effort to improve the reporting and monitoring of leishmaniasis in the Americas with the platform Sis-Leish, a recent analysis showed that approximately 50% of reported cases lacked data on clinical evolution^1, 2^.

In one Colombian reference center, sixty percent of CL patients who were not enrolled in any research study did not attend the follow-up visits after the formulation of antileishmanial medications (n=76). Among the 30 patients with at least one follow-up visit, 20% attended the end of treatment visit, 7% the visit at week 13, and 5% the visit at week 26^6^. The consensus for monitoring therapeutic response in cutaneous leishmaniasis is clinical evaluation of lesions, which is recommended at days 90 and 180 (weeks 13 and 26)^7^.

There are several challenges to the follow-up of patients with CL , such as limited access to health care services, long distances between rural communities and health care institutions, and resulting transportation costs^8^. Therefore, there is a need to develop alternative strategies for the follow-up of patients with CL in the rural areas of Colombia. Smartphone-based mHealth tools have shown promising results for surveillance, diagnosis and outcome assessment of infectious diseases in low-resource settings^9, 10^. The provision of mHealth tools to community health volunteers and rural health care workers for the follow-up of patients with CL in their communities, and the remote evaluation of lesions photographs by medical experts, could help to overcome some of the above challenges. Recently, a mobile application for therapy monitoring and assessment of therapeutic response in CL was developed, the Guaral+ST mobile app. Here we evaluated the validity of the Guaral+ST mobile application for remote assessment of therapeutic response in patients with CL using photographs captured by community health volunteers (CHV) with the app, compared to direct visualization of lesion healing by an expert physician.

## Materials and Methods

### Study design, population and setting

We conducted a validation study with prospectively and retrospectively recruited participants. The prospective enrolment of consecutive participants was performed at the CL referral clinics of CIDEIM in the municipalities of Tumaco and Cali in southwest Colombia. CIDEIM is a collaborating center of WHO for CL control. Tumaco is a mostly rural municipality which comprises 365 villages (*veredas*) and is endemic for CL. The city of Cali does not have local transmission of CL, but is where some patients from the Pacific Coast are treated.

Patients of any age, sex and ethnicity with parasitological diagnosis of CL were invited to participate in the study. They were excluded if muco-cutaneous or visceral leishmaniasis was suspected. Retrospective patients with parasitologically confirmed CL diagnosis were included if they had data on clinical response assessed by direct medical evaluation of the lesion, and if photographs were collected with the app at week 13 or later. Reporting of the study follows the Standard for the Reporting of Diagnostic Accuracy Studies (STARD 2015) guidelines (S1 table)^11^.

### Sample size calculation

For the purposes of our research, we considered that the Guaral+ST mobile app would be valid for the remote evaluation of patients if an agreement rate higher than 80% was achieved. We defined a sample size of 71 participants to estimate an agreement of 90% between the remote evaluation of therapeutic response through the photographs and performed directly by an expert physician, assuming 95% confidence and the width of the confidence interval of 7%^12^. For 95% confidence the corresponding Z statistic is 1.96, so the sample size was calculated as 1.962×0.9×(1-0.9)/0.072 = 71.

### The mobile app

The Guaral+ST mobile app for Android is designed to monitor CL treatment and assessment of therapeutic response. The app has two main functions: measure the side effects of pharmacologic treatment through periodic follow-up; and assess the treatment progress via photographic evaluation. The current study is restricted to the second function. Community leaders (Guaral+ST users) assess patients. They take pictures of a patient’s CL lesions periodically and the app uploads and saves the clinical photographs to cloud storage. Photographs were captured using a relatively low-cost smartphone (Motorola Moto G 5, 6, and 7). As patients are in areas with low or no internet coverage, the application stores data and photos in the device memory until the community leader can go to areas with internet coverage and upload the data to the cloud.

A second web-based desktop application called SND allows researchers and physicians the remote evaluation of therapeutic response. The web application allows access to the clinical photographs in order to be evaluated^13^. Access to both the mobile app and the web-based app were protected by password. The application ecosystem has an administrator role that allows registering new community leaders to access to the application and evaluate patients. Likewise, the administrator can register physicians or researchers to allow remote evaluation of the photographic archive.

The administrator, physicians and researchers can also assign patients to community leaders. Thus, community leaders only had access to the information of the participants evaluated by themselves. Data transfer is done by a secure channel through the HTTPS protocol (with TLS 1.3 encryption) and the photographic archive is stored in a secured server at a local university in Cali, Colombia (Universidad Icesi). The photographs (maximum 8 megapixels) are in PNG format to reduce transfer time. The server receives the data from the smartphones and sends it to SND for evaluation.

### App users and evaluators

Some app users were Community Health Volunteers (CHV) with a range of occupations^10^, while others were trained as nursing assistants (auxiliares de enfermería) and employed by the CL referral clinics in the municipalities of Tumaco and Cali. The evaluators were three physicians with different levels of experience in the care of CL patients: two had more than three years of experience, and the third had less than one.

### Training on how to use the app

The app users were trained in the use of the app and, in particular, the capture of photographs, during two 4-hour workshops before the field work. In the first workshop, the clinical aspects of CL were reviewed and the app users were trained in how to operate the Guaral+ST app. In the second workshop, app users were instructed on the capture of photographs with the app, including appropriate zoom, focus, illumination, and number of photographs per lesion.

Physicians were trained during two 3-hour workshops using clinical photographs of 20 cases with clinical cure and 20 cases with clinical failure. At the end of the second workshop a written examination was conducted to assure adequate understanding of the study and its evaluation procedures.

### Study procedures

#### Baseline evaluation

Following informed consent and clinical history taking, a physical examination was carried out by a physician with extensive experience in the care of CL cases. At least two photographs of each lesion were captured by the physician and treatment was prescribed according to Colombian guidelines^14, 15^. An electronic case report form (eCRF) was used to collect demographic and clinical information.

#### Follow-up

**Figure 1:**
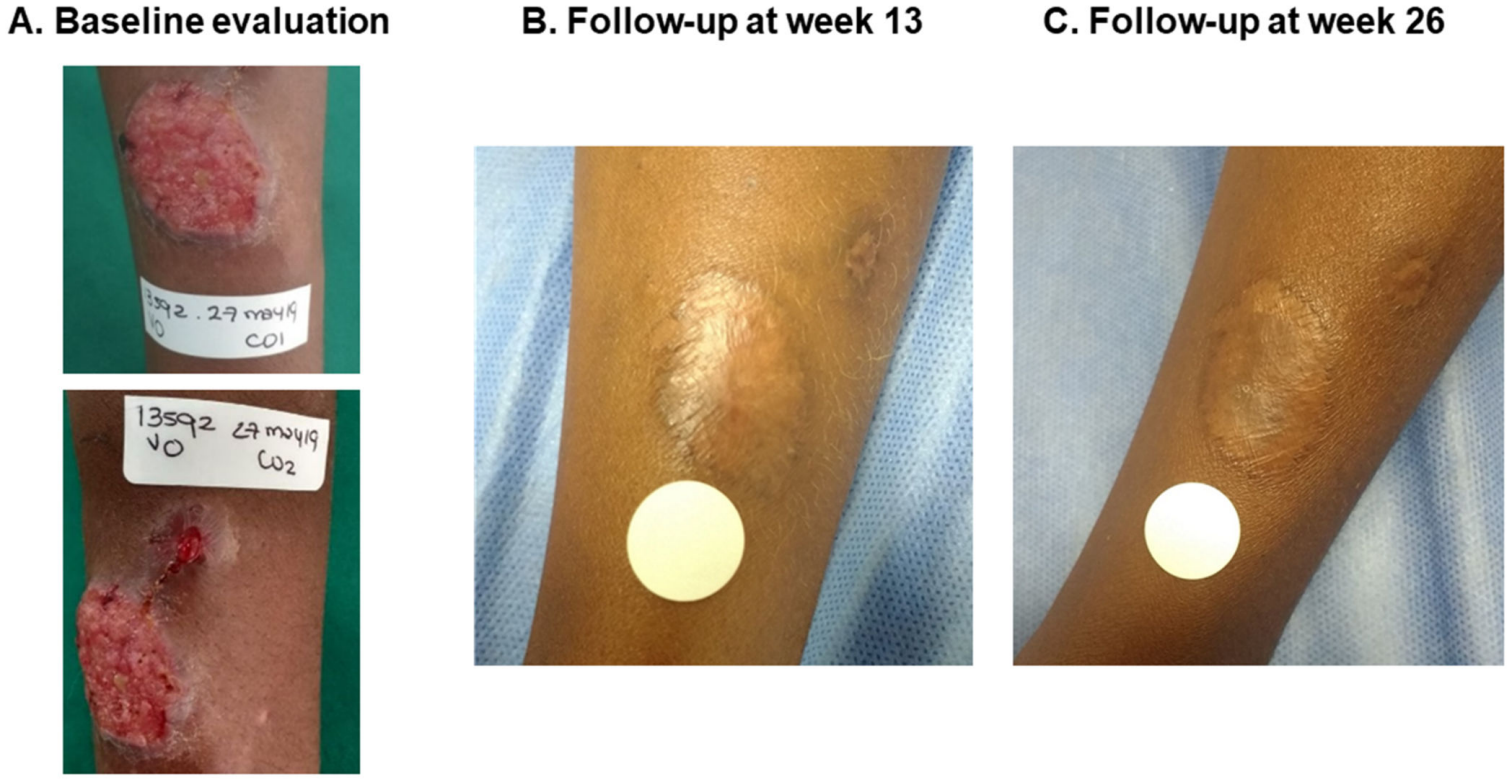
Photographs of a representative study participant. A 11 year-old participant with two typical cutaneous leishmaniasis lesions on the left arm. A. Baseline photographs captured by the study expert physician before treatment. B and C. Follow-up photographs captured using the Guaral+ST app. The participant was considered as having initial cure and definitive cure at weeks 13 and 26 respectively during the face-to-face medical evaluation. Only data for week 26 was included in the analysis.

Patients were evaluated by an expert physician for direct lesion assessment at weeks 13 (± 2 weeks) and 26 (± 4 weeks) to define the therapeutic response using the criteria proposed by Olliario et al.^7^. Additionally, two photographs of each lesion were captured with Guaral+ST by app mobile users. These photographs were taken on the same day as the medical evaluation or at most one week later (Fig 1).

#### Evaluation of lesion photographs

Photographs taken with the mobile app were evaluated by three trained physicians who did not participate in the care of the study participants. The ascertainment of therapeutic response (cure or failure) was independently assessed by each evaluator. Each rater compared the baseline and follow-up photographs. Applicable criteria (re-epithelization of ulcerated lesions, flattening of non-ulcerated lesions and characteristics of the ulcer border)^7^ were used to define cure or failure treatment. All evaluators were masked to each other and to the expert medical evaluation outcome (reference standard). Evaluation was by these human raters: there was no automated feature selection or extraction by computer.

In addition, the quality of each photograph (interpretable or uninterpretable) was assessed by each evaluator following the method described by Bowen et al.^16^, the parameters evaluated were magnification (too near, too far, or adequate), focus (lack of focus or adequate) and exposure (too light, too dark, or adequate).

### Index test

A summary evaluation of therapeutic response was generated based on the independent evaluation of the three physicians. To define cure or failure, at least two evaluators had to agree on the assessment.

### Reference standard

Direct medical evaluation of the lesions by an expert physician. Using the above criteria (including induration)^7^, initial cure or failure at week 13 and definitive cure or failure at week 26 was considered the reference standard. Currently, there are no other validated telemedicine-based or laboratory -based approaches to assess therapeutic response in CL.

### Statistical analysis

Data were extracted from SND and the eCRFs, and analyzed using the software Stata 16.0^17^. A descriptive analysis was conducted to identify the relative frequencies of gender, ethnicity, department and area of residence, as well as the median and range of age, number of lesions and time of evolution of lesions. When a participant attended both follow-up visits, the data of the later one were included in the analysis. Sensitivity, specificity, positive and negative predictive values were estimated between index (mobile app) and reference standard evaluations. The Wilson score method was used to calculate 95% confidence intervals for operating characteristics using the “diagt” command in Stata^18^. Concordance analysis between the summary evaluation of three physicians (index test), and direct lesion evaluation performed by the expert (reference standard) was done using Cohen’s kappa with its corresponding 95% CI, via the “kapci” command in Stata^19^. Kappa is a chance-adjusted measure of agreement: it measures observed agreement above chance, as a proportion of the maximum possible agreement above chance^20, 21^. Landis & Koch’s descriptors for values of kappa were used, e.g. values more than 0.8 are said to be “almost perfect”^22^. Finally, relative frequencies of the quality assessment of photographs were generated.

Considering the different level of experience of the evaluators, the operating characteristics and Cohen’s kappa were repeated considering the assessment of each evaluator in turn as the index evaluation.

### Ethics statement

This study was reviewed, approved and monitored by the institutional ethical review board of the Centro Internacional de Entrenamiento e Investigaciones Medicas (CIDEIM, reference number 1283). Written informed consent was obtained for all participants. Legal guardians provided written informed consent for minors (aged less than 18 years) and all minors aged 7 years or older gave written informed assent. Data from participants of a previous study who provided written informed consent for the use of their data in future studies were also included.

## Results

**Figure 2:**
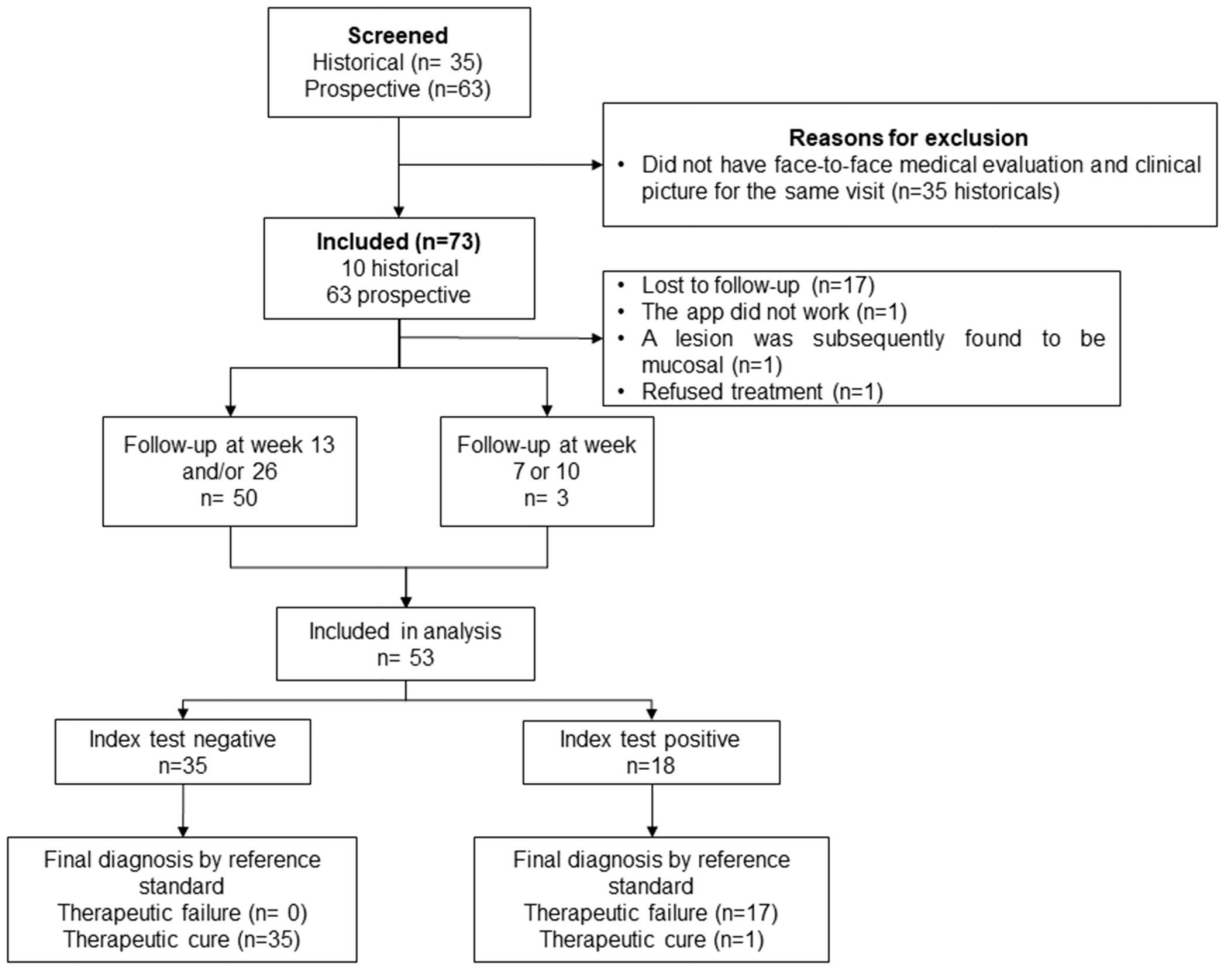
STARD diagram of the study participants. Flow diagram of the validation study. Shown all the eligible and recruited patients as well as reasons for exclusion of participants during the study.

Between September 19th 2018 and October 8th 2019, 63 participants with parasitological diagnosis of CL were invited to participate in the study and met eligibility criteria. Among the 35 historical participants identified from the previous study, only 10 met eligibility criteria, for a total of 73. However, 20 participants had to be excluded from the analysis, for reasons detailed in the flow diagram (Fig 2). Participants were mostly male (60.4%), of African descent (60.4%) and residents of rural areas (81.1%). Most participants had only ulcerated lesions (94.3%), approximately half participants had one lesion and three quarters received glucantime (Table 1). No adverse events related to the photography were reported to study personnel. Demographic and clinical characteristics of excluded and lost to follow-up participants are summarized in S2 Table. Those included in the analysis were generally similar to those who were excluded or lost to follow-up, in particular in terms of numbers and characteristics of lesions. However, a large majority (16/17) of those lost to follow-up were mestizo or indigenous, while these groups comprised only 40% of those included in the analysis.

### Validity of Guaral+ST app and agreement

Sensitivity and specificity for evaluation of the therapeutic response through photographs captured with the mobile app, compared with direct evaluation by an expert physician, were 100% (95%CI: 80.5%–100%) and 97.2% (95%CI 85.5–99.9) respectively. The agreement between evaluation of therapeutic response with photographs and direct evaluations was almost perfect (crude agreement=98.1%, kappa index=0.96, Table 2).

Although the sensitivity and positive predictive values were less than 90% for the evaluator with less than one year of experience (evaluator 3), agreement between remote and face-to-face assessment of therapeutic response remained “almost perfect” (kappa > 0.8) ^22^ when the analysis was stratified by evaluator. These analyses are presented in S3 and S4 tables.

### Quality assessment of photographs

Despite some clinical photographs (<10%) were considered uninterpretable, evaluators were able to assess therapeutic response for all participants, since at least two interpretable photographs were available for each lesion. Most photographs were considered as interpretable (>90% for each evaluator), with adequate focus (>70%) and adequate magnification (>69%). Evaluator 1 considered that only 45% of photographs had adequate exposure, but evaluators 2 and 3 considered most (>70%) had adequate exposure (Table 3). Most photographs considered uninterpretable had lack of focus (>90%, S5 Table).

## Discussion

Monitoring the effectiveness and safety of antileishmanial treatments is a priority for the control of CL in the Americas^8^. Such data for treatment administered within routine care are unavailable in Colombia and several countries of Latin America^1^. There is low adherence to clinical follow-up consultations, since 70-80% of the patients come from rural areas^2^, which limits their access to health services. mHealth tools for leishmaniasis can help close this knowledge gap and improve clinical management and epidemiological surveillance of leishmaniasis and other skin neglected tropical diseases^10, 23, 24, 25, 26^. This study reports the validation of the remote evaluation of therapeutic response in CL, using photographs captured by a mobile app. Our results show high sensitivity and specificity of this approach, which added to the use of relatively low-cost smartphones have the potential to improve timely identification and management of therapeutic failure. It could also help fill the gap on effectiveness data of CL therapy in rural, under-served populations ^8^. Successful implementation of such approaches depends on the training of app users in terms of the capture of good quality photographs, and at least two photographs per lesion. This, considering that lack of focus was the most common cause of photographs being uninterpretable (Table S4), which was overcome by taking two photographs per lesion.

Our results are in accordance with a growing body of evidence supporting the implementation of remote evaluation of photographs during the care of patients with dermatological conditions ^27^. Studies from high-income countries have found an agreement above 70% in the diagnosis of dermatological conditions using photographs versus direct medical evaluation and a concordance between 0.4 and 0.7 ^28, 29, 30^. Our higher agreement could be explained by the dichotomous nature of the outcome (cure and failure) and clear clinical criteria for its assessment. Likewise, our findings are consistent with the results of a previous study reporting the usefulness of smartphones for remote medical evaluation of images during infectious diseases consultation ^31^.

Limited connectivity and bandwidth are important challenges of using mHealth in remote settings ^32^. Therefore, a key component of the intervention is the capacity of the app to store information (including photographs) off-line until connectivity is available for synchronization. In addition, using an encrypted application such as Guaral+ST mobile app minimizes the risks regarding the protection of personal data of the participants compared to other more common alternatives such as instant messaging applications ^33^.

The remote evaluation of clinical photographs has been associated with lower costs for the health system and the patients, lower time to treatment and surgery for dermatological conditions and a reduction in the number of referrals ^34, 35^. However, this evidence comes from high income countries and a limited number of studies have also found an increase in the costs ^36, 37^. Therefore, the impact of the use of the Guaral+ST on the clinical outcome of patients with CL and the cost-effectiveness of this approach must be evaluated in further studies in rural settings.

Evidence of efficacy and effectiveness of antileishmanial treatments is limited due to the small sample size of clinical trials ^3^. A major limitation to reach large sample sizes in clinical trials in CL is the length of the follow-up requiring face-to-face medical evaluations at week 13 and 26 after treatment, which increases the costs for both the participants and the trial and precludes the inclusion of a substantial number of CL cases. Recently, Thomas et al included the remote evaluation of clinical photographs as the primary outcome in a clinical trial evaluating treatment for scabies ^38^. The implementation of the Guaral+ST app for outcome ascertainment in the design and conduct of clinical trials in CL may reduce trial cost, standardize the assessment of outcomes across several sites, and increase the sample size. The app can also register adverse events at the community level, and detect treatment failures early, hence providing information which is currently largely absent outside of clinical trial settings. The app can be used by community members which empowers local control over the disease.

Our study had some limitations. First, most participants included in our sample had only ulcerated lesions. Although this is typical of patients in the study area ^39, 40^, it meant that we were unable to produce reliable estimates of diagnostic performance parameters for other type of lesions and our results may not be applicable to patients with non-ulcerated lesions. Furthermore, while most patients with CL have ulcerated lesions, the main criteria for assessment of therapeutic response in non-ulcerated lesions are flattening of lesions, which may be more difficult to assess through photographs and induration that can be assessed only through physical examination ^7^. We had information on the lesions at baseline of those who were excluded from the study, or lost to follow-up, and these were similar to those who were included in the analysis. So selection bias does not seem to be a major concern. In addition, while we included in our sample a broadly representative sample of CL cases in Colombia, we were unable to reach the target sample size due to losses to follow-up. We assess this limitation in terms of the confidence limits of the main parameters ^41^, and note that the lower limits of both sensitivity and specificity are above 80%, so we consider that the results are still informative. In terms of losses to follow-up, such a status may have been associated with cure, although the association could plausibly be either positive (no perceived need for further visits) or negative (disillusionment). Losses to follow-up might have been reduced if it had been possible to have versions of the app in indigenous languages. Another limitation is that we analyzed at the level of individual evaluator, as well as at the summary level, even though the former was not included in the statistical analysis plan. Most app users had a relatively high level of education (nursing assistants). However, other app users had different occupations and secondary school level of education, and were still able to easily capture photographs after short training. The number of physicians (three) was small, which may limit the generalizability of the findings. On the other hand, all physicians examined all patients, so clinical differences between patients, e.g., in treatment, cannot explain between-physician differences in results. Our study had several further strengths, including the standardized training on the use of the app, the capture of clinical photographs by the app users, and the evaluation of clinical photographs by physicians. We used mid-range smartphone models (Moto G 6 and 7) so, for future use, image quality is not likely to be a constraint. Moreover, smartphones are widely used in Colombia: as of 2020^42^, 79% of the total population of Colombia had a mobile connection, with 67% of these connections using smartphones. Community health workers with varying degrees of education, including some with only elementary school, were successfully trained in eight hours. Longer training on practical aspects of the app and periodic re-training workshops could help increase the quality of the pictures.

Our results support the use of the Guaral+ST app for the capture of photographs of CL lesions for the remote evaluation of therapeutic response by experienced clinicians. We are currently evaluating barriers and facilitators to the scaling-up of the app, in particular its integration with existing health information systems. This app has the potential to help overcome the knowledge gap about the real-world effectiveness of antileishmanial therapy, mainly in rural areas. Additionally, it could support the design of larger clinical studies to assess effectiveness of antileishmanials, and enhance the timeliness of identification and access to rescue treatment for cases with therapeutic failure.

## Supplementary Material

Supplementary information

## Figures and Tables

**Table 1:** Clinical and demographic characteristics of the study population

Characteristics	Historical n=10	Prospective n=43	Total n=53
Median age in years (range)	27.5 (8.2-53.3)	25.8 (0.7-69.2)	25.8 (0.7-69.2)
Male sex n (%)	7 (70.0)	25 (58.1))	32 (60.4)
Ethnicity			
Afro-Colombian n (%)	9 (90.0)	23 (53.5)	32 (60.4)
*Mestizo* n (%)	1 (10.0)	13 (30.2)	14 (26.4)
Indigenous n (%)	0	7 (16.3)	7 (13.2)
Department of residence			
Nariño n (%)	10 (100)	31 (72.1)	41 (77.4)
Valle del Cauca n (%)	0	5 (11.6)	5 (9.4)
Other n (%)	0	7 (16.3))	7 (13.2)
Area of residency			
Rural n (%)	8 (80.2)	35 (81.4)	43 (81.1)
Urban n (%)	2 (20.0)	8 (18.6)	10 (18.9)
Treatment			
Glucantime (%)	9 (90)	31 (72.1)	40 (75.5)
Miltefosine (%)	1 (10)	12 (27.9)	13 (24.5)
Type of lesions			
All ulcerated (%)	10 (100)	40 (93.0)	50 (94.3)
Non-ulcerated (%)	0	0	0
Mixed (%)	0	3 (7.0)	3 (5.7)
Median time of evolution of lesions in months (range)	2.5 (0.5-9)	1.5 (0.5-5)	1.5 (0.5-9)
Median number of lesions (range)	1.5 (1-3)	1 (1-6)	1 (1-6)

**Table 2:** Validity of Guaral+ST app and agreement between assessment of therapeutic response with photos and direct medical evaluation

	**Direct evaluation of therapeutic response**		
**Therapeutic response evaluated by photographs: summary over the three evaluators**	Failure	Cure	**Total**
Failure	17	1	18
Cure	0	35	35
**Total**	17	36	53
**Validity % (95% CI)**			
Sensitivity	100 (80.5 - 100)		
Specificity	97.2 (85.5 – 99.9)		
Positive predictive value	94.4 (72.7 – 99.9)		
Negative predictive value	100 (90.0-100)		
**Agreement %**	98.1%		
**Kappa index (95% CI)**	0.96 (0.88 – 1.00)		

**Table 3:** Quality assessment of photographs collected by the mobile application

	Evaluator 1	Evaluator 2	Evaluator 3
Number of photographs assessed	191	189	192
**Overall quality assessment**			
Interpretable n (%)	182 (95.3)	174 (92.1)	187 (97.4)
Uninterpretable n (%)	9 (4.7)	15 (7.9)	5 (2.6)
**Focus**			
Adequate n (%)	149 (78.0)	146 (77.3)	153 (79.3)
Lack of focus n (%)	42 (22.0)	43 (22.8)	40 (20.7)
**Magnification**			
Adequate n (%)	141 (73.8)	131 (69.3)	161 (83.4)
Too near n (%)	8 (4.2)	3 (1.6)	6 (3.1)
Too far n (%)	42 (22.0)	55 (29.1)	26 (13.5)
**Exposure**			
Adequate n (%)	84 (45.0)	139 (73.5)	150 (77.7)
Too light n (%)	31 (16.2)	38 (20.1)	15 (7.8)
Too dark n (%)	76 (39.8)	12 (86.5)	28 (14.5)
