# Supplementary material for: Sensitivity and specificity of the remote evaluation of therapeutic response in cutaneous leishmaniasis using photographs from a mobile application
Source: Am J Trop Med Hyg. Author manuscript; Available in PMC 2023 Mar 10. (PMC9490675; doi:10.4269/ajtmh.22-0164)
Supplement: Supplementary information [file EMS153398-supplement-Supplementary_information.docx]

**Supporting information**

**S1 Table. STARD Checklist**

|  | **Section & Topic** | **No** | **Item** | **Reported on page #** |
| --- | --- | --- | --- | --- |
|  |  |  |  |  |
|  | **TITLE OR ABSTRACT** |  |  |  |
|  |  | **1** | Identification as a study of diagnostic accuracy using at least one measure of accuracy  (such as sensitivity, specificity, predictive values, or AUC) | 1 |
|  | **ABSTRACT** |  |  |  |
|  |  | **2** | Structured summary of study design, methods, results, and conclusions  (for specific guidance, see STARD for Abstracts) | 2 |
|  | **INTRODUCTION** |  |  |  |
|  |  | **3** | Scientific and clinical background, including the intended use and clinical role of the index test | Third paragraph of introduction |
|  |  | **4** | Study objectives and hypotheses | Third paragraph of introduction |
|  | **METHODS** |  |  |  |
|  | *Study design* | **5** | Whether data collection was planned before the index test and reference standard  were performed (prospective study) or after (retrospective study) | First sentence of “Study design and population” sub-heading of the methods section |
|  | *Participants* | **6** | Eligibility criteria | “Study design and population” sub-heading of the methods section |
|  |  | **7** | On what basis potentially eligible participants were identified  (such as symptoms, results from previous tests, inclusion in registry) | “Study design and population” sub-heading of the methods section |
|  |  | **8** | Where and when potentially eligible participants were identified (setting, location and dates) | “Study design and population”, and “setting” sub-headings of the methods section and first paragraph of the results section |
|  |  | **9** | Whether participants formed a consecutive, random or convenience series | Second sentence of “study design and population” sub-heading of the methods section |
|  | *Test methods* | **10a** | Index test, in sufficient detail to allow replication | “Evaluation of lesion photographs” and “index test” subheadings of the methods section. |
|  |  | **10b** | Reference standard, in sufficient detail to allow replication | “Follow-up evaluations” and “reference standard” sub-headings of the methods section. |
|  |  | **11** | Rationale for choosing the reference standard (if alternatives exist) | Last sentence of the “reference standard” sub-heading of the methods section |
|  |  | **12a** | Definition of and rationale for test positivity cut-offs or result categories  of the index test, distinguishing pre-specified from exploratory | The applicable components of the widely accepted applicable Olliaro et al. criteria were used. |
|  |  | **12b** | Definition of and rationale for test positivity cut-offs or result categories  of the reference standard, distinguishing pre-specified from exploratory | The widely accepted Olliaro criteria were used. |
|  |  | **13a** | Whether clinical information and reference standard results were available  to the performers/readers of the index test | Last sentence of the first paragraph of the “evaluation of lesion photographs” sub-heading of the methods section |
|  |  | **13b** | Whether clinical information and index test results were available  to the assessors of the reference standard | “Follow-up evaluations” sub-heading of the methods section. |
|  | *Analysis* | **14** | Methods for estimating or comparing measures of diagnostic accuracy | “Statistical analysis” sub-heading of methods section |
|  |  | **15** | How indeterminate index test or reference standard results were handled | Not applicable because even though some photographs were not evaluable, all lesions had at least one evaluable photograph. |
|  |  | **16** | How missing data on the index test and reference standard were handled | Participants without data on the reference standard (those lost to folloy-up) were excluded. See the figure 1 |
|  |  | **17** | Any analyses of variability in diagnostic accuracy, distinguishing pre-specified from exploratory | Last sentence of the “Statistical analysis” subsection of the methods section. |
|  |  | **18** | Intended sample size and how it was determined | “Sample size calculation” sub-heading of the methods section |
|  | **RESULTS** |  |  |  |
|  | *Participants* | **19** | Flow of participants, using a diagram | Figure 1. STARD diagram |
|  |  | **20** | Baseline demographic and clinical characteristics of participants | Table 1 |
|  |  | **21a** | Distribution of severity of disease in those with the target condition | Table 1 |
|  |  | **21b** | Distribution of alternative diagnoses in those without the target condition | Not applicable because all participants had confirmed leishmaniasis and the outcome is the therapeutic outcome. |
|  |  | **22** | Time interval and any clinical interventions between index test and reference standard | Last sentence of “follow-up evaluations” sub-heading of the methods section |
|  | *Test results* | **23** | Cross tabulation of the index test results (or their distribution)  by the results of the reference standard | Table 2 |
|  |  | **24** | Estimates of diagnostic accuracy and their precision (such as 95% confidence intervals) | Table 2 |
|  |  | **25** | Any adverse events from performing the index test or the reference standard | First paragraph of Results section. |
|  | **DISCUSSION** |  |  |  |
|  |  | **26** | Study limitations, including sources of potential bias, statistical uncertainty, and generalisability | Fifth paragraph of the discussion section |
|  |  | **27** | Implications for practice, including the intended use and clinical role of the index test | First and second paragraph of the discussion. |
|  | **OTHER INFORMATION** |  |  |  |
|  |  | **28** | Registration number and name of registry | Not available |
|  |  | **29** | Where the full study protocol can be accessed | Not available |
|  |  | **30** | Sources of funding and other support; role of funders | Funding section |
|  |  |  |  |  |

| **S2 Table. Clinical and demographic characteristics of subjects who were excluded, lost to follow-up and included in the analysis.** | | | |
| --- | --- | --- | --- |
| **Characteristics** | **Excluded**  n=28* | **Lost to follow-up**  n=17 | **Included in the analysis**  n=53 |
| Median age in years (range) | 26.3 (2.3-78) | 28 (1-61) | 25.8 (0.7-69.2) |
| Male sex n (%) | 17 (60.7) | 12 (70.5) | 32 (60.4) |
| Ethnicity |  |  |  |
| Afro-Colombian n (%) | 14 (50.0) | 1 (5.9) | 32 (60.4) |
| *Mestizo* n (%) | 8 (28.6) | 9 (52.9) | 14 (26.4) |
| Indigenous n (%) | 6 (21.4) | 7 (41.2) | 7 (13.2) |
| Department of residence |  |  |  |
| Nariño n (%) | 26 (92.9) | 11 (64.7) | 41 (77.4) |
| Valle del Cauca n (%) | 1 (3.6) | 2 (11.8) | 5 (9.4) |
| Other n (%) | 1 (3.6) | 4 (23.5) | 7 (13.2) |
| Area of residency |  |  |  |
| Rural n (%) | 22 (78.6) | 13 (76.5) | 43 (81.1) |
| Urban n (%) | 6 (21.4) | 4 (23.5) | 10 (18.9) |
| Treatment |  |  |  |
| Glucantime (%) | 24 (85.7) | 11 (64.7) | 40 (75.5) |
| Miltefosine (%) | 4 (14.3) | 6 (35.3) | 13 (24.5) |
| Type of lesions |  |  |  |
| All ulcerated (%) | 25 (89.3) | 16 (94.1) | 50 (94.3) |
| Non-ulcerated (%) | 3 (10.7) | 1 (5.1) | 0 |
| Mixed (%) | 0 | 0 | 3 (5.7) |
| Median time of evolution of lesions in months (range) | 1.5 (0.5-6) | 1 (1-4) | 1.5 (0.5-9) |
| Median number of lesions (range) | 2 (1-9) | 1 (1-4) | 1 (1-6) |
| Therapeutic response |  |  |  |
| Cure | 19 (67.9) | 0 | 35 (66.0) |
| Failure | 7 (25.0) | 0 | 18 (34.0) |
| Unknown | 2 (7.1) | 17 (100) | 0 |
| * 3 prospectives and 25 historical CL cases. | | | |

| **S3 Table. Operating characteristics of remote evaluation of therapeutic response related to face-to-face medical evaluation by evaluator** | | | | |
| --- | --- | --- | --- | --- |
|  | **Sensitivity**  **%**  **(95%CI)** | **Specificity**  **%**  **(95%CI)** | **Positive predictive value**  **%**  **(95%CI)** | **Negative predictive value**  **%**  **(95%CI)** |
| **Evaluator 1** | 94.1  (71.3-99.9) | 97.2  (85.5-99.9) | 94.1  (71.3-99.9) | 97.2  (85.5-99.9) |
| **Evaluator 2** | 100  (80.5-100) | 94.1  (80.3-99.3) | 89.5  (66.9-98.7) | 100  (89.1-100) |
| **Evaluator 3** | 82.4  (56.6-96.2) | 94.3  (80.8-99.3) | 87.5  (61.9-98.4) | 91.7  (77.5-98.2) |

| **S4 Table. Agreement of remote evaluation of therapeutic response related to face-to-face medical evaluation by evaluator** | | | | | |
| --- | --- | --- | --- | --- | --- |
|  | **Face-to-face evaluation of therapeutic response** | | | **Agreement**  **%** | **Kappa**  **(95%CI)** |
| **Remote evaluation of therapeutic response by evaluator 1** | Failure | Cure | **Total** | 96.2 | 0.91  (0.80-1.00) |
| Failure | 16 | 1 | 17 |  |  |
| Cure | 1 | 35 | 36 |  |  |
| **Total** | 17 | 36 | 53 |  |  |
| **Remote evaluation of therapeutic response by evaluator 2** |  |  |  | 96.1 | 0.91  (0.80-1.00) |
| Failure | 17 | 2 | 19 |  |  |
| Cure | 0 | 32 | 32 |  |  |
| **Total** | 17 | 34 | 51 |  |  |
| **Remote evaluation of therapeutic response by evaluator 3** |  |  |  | 90.4 | 0.78  (0.60-0.96) |
| Failure | 14 | 2 | 16 |  |  |
| Cure | 3 | 33 | 36 |  |  |
| **Total** | 17 | 35 | 52 |  |  |
| **Agreement between the three evaluators** | | | | - | 0.77  (0.69-0.84) |

| **S5 Table. Characteristics of clinical pictures considered as uninterpretable** | | | |
| --- | --- | --- | --- |
|  | **Evaluator 1**  n=9 | **Evaluator 2**  n=15 | **Evaluator 3**  n=5 |
| **Focus** |  |  |  |
| Adequate n (%) | 0 | 2 (13.3) | 0 |
| Lack of focus n (%) | 9 (100) | 13 (86.7) | 5 (100) |
| **Magnification** |  |  |  |
| Adequate n (%) | 4 (44.4) | 9 (60.0) | 1 (20.0) |
| Too near n (%) | 1 (11.1) | 0 | 1 (20.0) |
| Too far n (%) | 4 (44.4) | 6 (40.0) | 3 (60.0) |
| **Exposure** |  |  |  |
| Adequate n (%) | 1 (11.1) | 11 (73.3) | 1 (20) |
| Too light n (%) | 1 (11.1) | 2 (13.3) | 0 (0) |
| Too dark n (%) | 7 (77.8) | 2 (13.3) | 4 (80) |
